# Electronic Structure, Lattice Dynamics, and Pressure-Induced Phase Transitions in Gd_2_MoO_6_: A Combined Theoretical and Experimental Study

**DOI:** 10.1021/acsomega.5c11574

**Published:** 2026-02-10

**Authors:** Danilo S. Luz, Luiz F. L. da Silva, Raí F. Juca, Vicente O. Sousa Neto, Antônio J. Ramiro de Castro, Francisco F. de Sousa, Waldeci Paraguassu, Rômulo S. Silva, Lucas S. A. Olivier, José A. Lima, Paulo de T. C. Freire, João G. de Oliveira Neto, Gilberto D. Saraiva

**Affiliations:** † Center for Social Sciences, Health, and Technology, Federal University of Maranhão, Imperatriz, Maranhão 65900-410, Brazil; ‡ Criminalistics Institute, Scientific Police of Pará, Marabá, Pará 68507-000, Brazil; § Faculty of Education, Sciences and Letters of the Sertão Central, State University of Ceará, Quixadá, Ceará 63902-098, Brazil; ∥ Federal University of Ceará, Cedro, Quixadá, Ceará 63902-580, Brazil; ⊥ Institute of Exact and Natural Sciences, 37871 Federal University of Para, Belém, Pará 66075-110, Brazil; # Department of Physics, Federal University of Ceará, Fortaleza, Ceará 60455-970, Brazil

## Abstract

This study presents a combined theoretical and experimental investigation into the structural, electronic, and vibrational properties of Gd_2_MoO_6_. To gain deeper insight into its chemical composition, first-principles calculations were employed, emphasizing energy band analysis. The conduction band minimum is positioned at the high-symmetry Γ-point, while the valence band maximum appears between the Z and Γ-points. These results indicate that Gd_2_MoO_6_ is a semiconductor exhibiting an indirect band gap of approximately 1.92 eV. Furthermore, lattice dynamics were examined using density functional theory (DFT) to interpret the experimental Raman and infrared spectra. Hirshfeld surface and structural analyses reveal that Gd_2_MoO_6_ exhibits a hybrid ionic-covalent framework governed by dominant Gd–O/Gd–O and Mo–O/O–Mo bonds. Additionally, pressure-dependent Raman spectroscopy was carried out to explore structural modifications resulting from pressure variations. Based on the spectral changes, two phases’ transitions were identified at approximately 3.1–3.3 GPa and 9.5–10 GPa, potentially linked to increased disorder of octahedra induced by pressure effects. The principal component analysis and hierarchical cluster analysis identified two phase transitions at near pressure range of 3.1–3.3 GPa and 9.5–10. GPa, which are in agreement with the pressure-dependent Raman studies.

## Graphical abstract

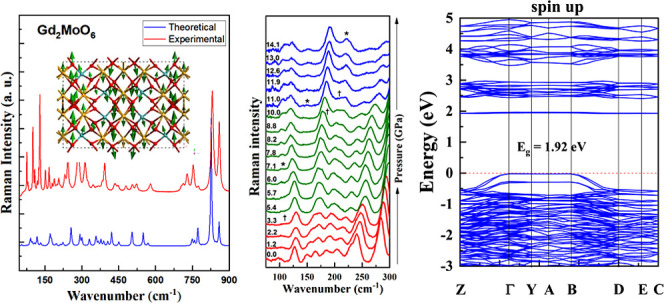

## 1 Introduction

Rare-earth molybdates (RE), represented by the chemical formula RE_2_MoO_6_ (where RE corresponds to a rare-earth ion), form a fascinating family of materials whose chemical and physical properties can be tailored through the appropriate selection of RE cations.^1–3^ These compounds are widely recognized for their remarkable characteristics, including luminescence,^4–7^ versatile fluorescent properties,^8^ thermal stability,^9,10^ photodegradation,^11^ electrocatalytic oxygen evolution,^12^ magnetocaloric effects,^13–16^ and more.^6,17–21^ Their primary field of study lies on their utilization as phosphors, serving as luminescent matrices for trivalent RE ions such as Yb^3+^, Eu^3+^, Sm^3+^, Tm^3+^, Ho^3+^, Dy^3+^, Tb^3+^, and others, with their spectroscopic properties being extensively investigated owing to their potential in optics.^22–26^ When doped with RE ions such as Er^3+^, Yb^3+^, or Nd^3+^, gadolinium molybdates demonstrate enhanced solid-state laser performance in the infrared and visible ranges.^3^ Specifically, Gd_2_(MoO_4_)_3_ doped with Eu^3+^, Tb^3+^, or Dy^3+^ has been extensively studied as a potential phosphor candidate for white light-emitting diodes (LEDs) and display backlighting, due to its high quantum efficiency, thermal stability, and tunable emission spectrum.^4^ Beyond their photoluminescent properties, gadolinium (Gd)-based compounds have been widely studied in bioimaging and multiple sclerosis research, further motivating in-depth investigations into these materials.^27,28^

Among Gd-based molybdates, Gd_2_MoO_6_ has attracted increasing attention due to its multifunctional properties that extend well beyond its ease of synthesis. Recent studies have demonstrated that Gd_2_MoO_6_ exhibits efficient down-conversion and up-conversion luminescence, making it suitable for advanced optical devices and photonic applications.^29^ Its optical response and ferroelectric-related behavior have also been investigated, highlighting its potential for device-oriented applications.^30^ Furthermore, Gd-containing oxides, including Gd_2_MoO_6_-based systems, have been explored for biomedical applications such as bioimaging and contrast agents, owing to the favorable magnetic and optical characteristics of Gd^3+^ ions.^31–33^ In particular, Eu^3+^-activated Gd_2_MoO_6_ phosphors have shown intense red emission under near-ultraviolet and blue excitation, demonstrating strong potential for LEDs and solid-state lighting technologies.^34^ These results establish Gd_2_MoO_6_ as a versatile functional material whose optical, electronic, and magnetic properties are highly sensitive to its local structure and external perturbations.

In addition, these materials exhibit a wide range of functional properties that can be strategically modified or tuned under varying experimental conditions. Among these approaches, high-pressure techniques enable a detailed analysis of subtle changes in the crystal lattice that influence these properties, while studies at low temperatures help in understanding many physical phenomena.^35–37^ These studies can provide important information on the structural aspects of compounds, which in turn provides deeper insight into the fundamental phenomena governing characteristics such as phonon anharmonicity,^38^ negative thermal expansion.^39,40^ ferroelectric,^41^ structural phase transitions,^42,43^ and pressure-induced amorphization.^44–46^ Studies on Gd_2_(MoO_4_)_3_ indicate that this material undergoes several structural transitions between 2 and 6 GPa, with amorphization occurring at higher pressures. These transitions are associated with the reorganization of MoO_4_ tetrahedra and changes in molybdenum coordination.^47^ Due to their pressure-sensitive Raman modes, Gd_2_(MoO_4_)_3_ materials have been explored as candidates for pressure-sensing and piezoelectric applications, where phase stability under extreme conditions is crucial.^44–47^ Studies have demonstrated that external pressure induces structural transformations, influencing the vibrational properties of MoO_4_ tetrahedra and making these materials promising candidates for pressure-driven phase-change applications.^35,36,44–46^

Thus, the monoclinic phase of Gd_2_MoO_6_, belonging to the *C*_2_/*c* space group, is of particular interest due to its easy synthesis and the limited investigation of its behavior in Raman spectroscopy under high pressure. In this work, we explore the properties of Gd_2_MoO_6_ under high pressure using Raman spectroscopy. Initially, the electronic band structure and partial density of states (PDOS) are analyzed through first-principles calculations. Then, the nature of the observed vibrational modes is explored at the same theoretical level, allowing for the prediction of wavenumbers, mode assignments, and symmetry of each Raman-active mode. Finally, Raman spectra under high pressure are examined, unveiling changes in the band profile and indicating a potential phase transition.

## 2 Experimental Methodology

Polycrystalline Gd_2_MoO_6_ samples were synthesized by using the solid-state reaction method, which involves homogenizing precursor powders. Initially, the starting materials were manually ground for 30 min using a mortar and pestle to ensure uniform mixing. The resulting mixture was then subjected to heat treatment at 950 °C for 24 h in an ambient atmosphere using a muffle-type resistance furnace, with a controlled heating rate of 10 °C/min. The Gd_2_MoO_6_ sample was synthesized in a 1:1 molar ratio from gadolinium­(III) oxide (Gd_2_O_3_, 99.99% purity, Aldrich Chemicals Ltd.) and molybdenum trioxide (MoO_3_, 99.99% purity, Aldrich Chemicals Ltd.), as represented in eq 1. The synthesized sample was allowed to cool to room temperature naturally, utilizing the thermal inertia of the furnace, crucible, and sample assembly.

1 $${Gd}_{2}O_{3\left(s\right)}+{MoO}_{3\left(s\right)}\to {Gd}_{2}{MoO}_{6\left(S\right)}$$

The synthesized sample was characterized by X-ray diffraction (XRD) through a powder diffraction method on a PANalytical Empyrean diffractometer. The instrument operated with Cu Kα radiation (λ = 1.5418 Å) in Bragg–Brentano geometry and featured a pyrolytic graphite monochromator at room temperature. Diffraction data were recorded over a 2θ range of 10°–80°, with a step size of 0.02° and a counting time of 2.0 s per step. The GSAS-I^48^ software was used for Rietveld refinement, utilizing crystal data from the Inorganic Crystal Structure Database (ICSD), Card No. 32173.^49^ Morphological analyses were carried by means of a high-resolution scanning electron microscopy (SEM), model Vega3 SBH from TESCAN, equipped with a Bruker Xflash 410 M energy-dispersive spectrometer (EDS). The absorption spectrum of the Gd_2_MoO_6_ sample was obtained by using a PerkinElmer Frontier Fourier transform infrared (FTIR) spectrophotometer. Measurements were performed in the spectral range of 4000–400 cm^–1^ with a resolution of 2 cm^–1^, averaging 32 scans. The analysis utilized an attenuated total reflectance (ATR) accessory with a germanium crystal. Raman measurements were performed using a Horiba T64000 spectrometer equipped with a liquid N_2_-cooled CCD system. This system was integrated with the following components: (i) a 532 nm argon ion laser, (ii) an Olympus microscope lens with a focal length of 20.5 mm and a numerical aperture (NA) of 0.35 for laser focusing on the sample surface, and (iii) a spectrometer slit providing a resolution of approximately 2 cm^–1^. For pressure-dependent measurements, a diamond anvil cell (DAC) was employed, utilizing a mixture of methanol–ethanol (4:1) as the pressure-transmitting medium over a range of 0.0–14.1 GPa. Pressure calibration within the sample chamber, which consists of a hole in a stainless-steel gasket positioned between two diamond anvils, was performed using the luminescence of Cr^3+^ ions in ruby.

## 3 Theoretical Calculations

The noncovalent interactions and voids crystal calculations were performed employing CrystalExplorer 17.^50^ For a comprehensive analysis of molecular contacts, we generated three-dimensional Hirshfeld surfaces with normalized contact distance (*d*_norm_) mapping, derived from the external (*d*_e_) and internal (*d*_*i*_) distances relative to van der Waals radii (*r*_vdW_).^51^ This approach enabled both qualitative visualization and quantitative assessment of various weak interactions in the crystal structure. Furthermore, we characterized the void spaces within the primitive unit cell through procrystal electron density analysis, using an isosurface threshold of 0.02 atomic units for accurate void quantification.^52^

The electronic and vibrational properties of Gd_2_MoO_6_ were investigated using Density Functional Theory (DFT) as implemented in the Cambridge Serial Total Energy Package (CASTEP).^53^ Norm-conserving pseudopotentials were employed to represent the core electrons,^54^ while the exchange–correlation effects were treated within the Generalized Gradient Approximation (GGA) using the Perdew–Burke–Ernzerhof (PBE) functional. Brillouin zone integrations were performed using a 2 × 2 × 3 Monkhorst–Pack *k*-point mesh.^55^ A fine energy cutoff of 820 eV was used for the plane-wave basis set, ensuring well-converged results. To account for the strong on-site Coulomb interactions of the localized *d* and *f* electrons, particularly those of Gd and Mo, the DFT + *U* approach was employed.^56,57^ The Hubbard U parameter, which effectively corrects for these interactions, was applied to the relevant atomic orbitals. The atomic positions were optimized using the Broyden-Fletcher-Goldfarb-Shanno (BFGS) algorithm^58^ until the following convergence criteria were met: a maximum energy change of 1.0 × 10^–6^ eV/atom, a maximum force of 0.03 eV/Å, a maximum stress of 0.1 GPa, and a maximum displacement of 0.001 Å. Following structural optimization, the electronic band structure and associated properties were computed by propagating the electronic wave function along high-symmetry points in the Brillouin Zone (BZ). The chosen *k*-point path was *Z* (0.000, 0.000, 0.500); Γ (0.000, 0.000, 0.000); *Y* (0.000, 0.500, 0.000); A (−0.500, 0.500, 0.000); B (−0.500, 0.000, 0.000); D (−0.500, 0.000, 0.500); E (−0.500, 0.500, 0.500); and C (0.000, 0.500, 0.500). The calculations were performed on a monoclinic cell (space group *C*_2_/*c*) containing a total of 78 atoms.

The determination of vibrational frequencies involves calculating the spatial derivatives of macroscopic polarization, as detailed by Porezag and Pederson. These derivatives are numerically computed along the eigenvectors of each Raman-active phonon mode using the linear response formalism to determine the polarization for each displacement.^59^ Consequently, the Raman susceptibility tensor, which is fundamental for calculating Raman intensity, is established in eq 2.

2 $$A_{\alpha \beta }^{m}=\sqrt{V}\sum_{I\gamma }\frac{{d\chi }_{\alpha \beta }^{\left(1\right)}}{{dR}_{I\gamma }}\frac{\upsilon_{I\gamma }^{m}}{\sqrt{M_{I}}}$$

where χ_αβ_^(1)^ is the first order dielectric susceptibility and υ is the phonon eigenvector in the direction in which the atom *I*, of mass *M*_I_ at equilibrium positions *R*, move under excitation of a phonon mode (*m*), in a unit cell with volume *V*.^56^

## 4 Results and Discussion

### 4.1 Crystal Structure Analysis

Figure 1 shows the powder XRD pattern refined through the Rietveld method using GSAS I software to determine the structural parameters. The XRD pattern reveals that Gd_2_MoO_6_ crystals crystallize in a monoclinic symmetry with space group *C*_2_/*c* (*C*_2*h*_^6^, No. 15), containing eight molecular formula per unit cell (*Z* = 8). The unit cell refined parameters are *a* = 16.517(3) Å, *b* = 11.185(5) Å, *c* = 5.424(4) Å, with α = γ = 90.00°, β = 108.30(5) °, resulting in a *V* of 951.523(8) Å^3^, values that are very close to those reported by others.^6,60^ The fitting parameters, *R*_p_ = 4.48%, *R*_wp_ = 5.83%, and χ^2^ = 1.02, indicate good agreement between the calculated and experimental XRD pattern. A schematic representation of the primitive unit cell of Gd_2_MoO_6_ crystals is shown in Figure 2, where each Gd^3+^ cation is coordinated by four oxygen eight atoms occupying octahedral sites, while the Mo^6+^ atoms are bonded to five oxygen atoms, forming a polyhedron.

**Figure 1:**
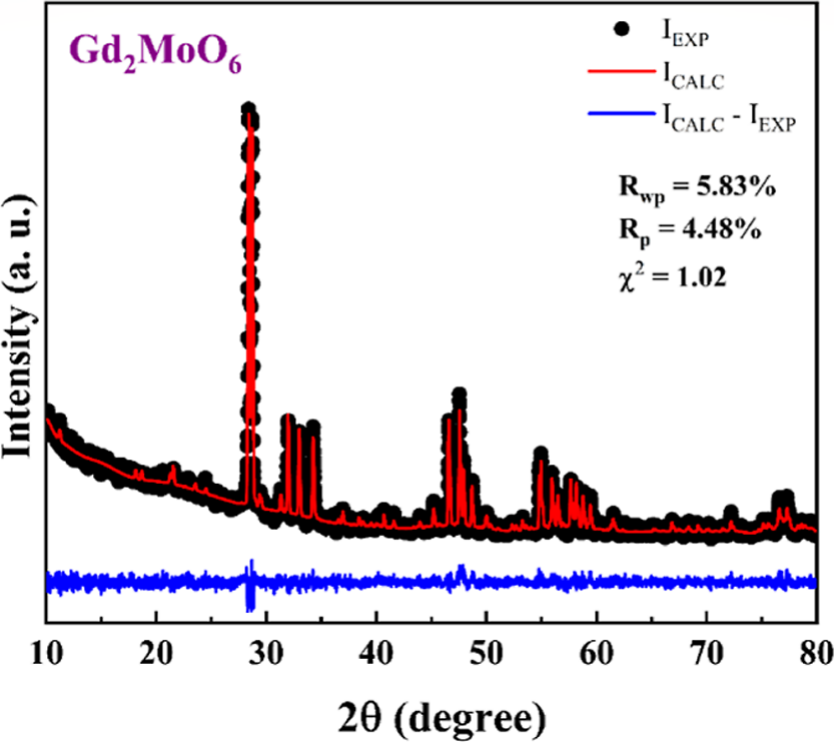
Rietveld refinement of the powder XRD data for Gd_2_MoO_6_; the black circles and red lines represent the experimental pattern and its refinement, respectively.

**Figure 2:**
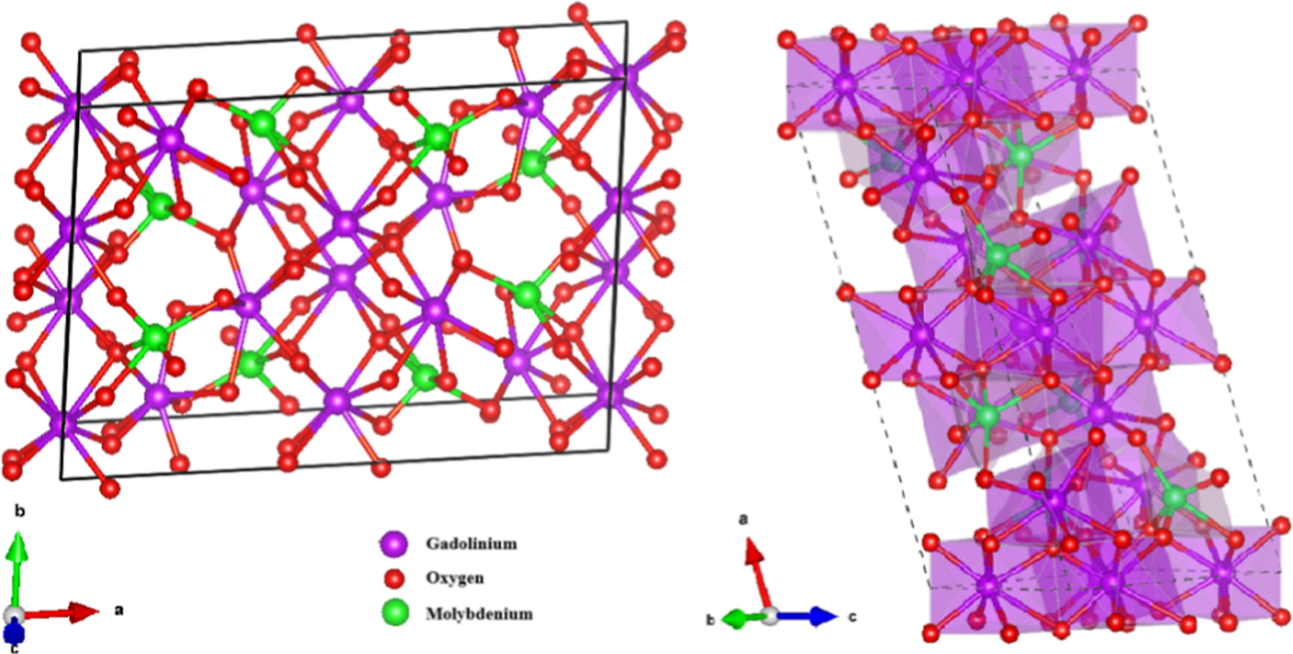
A primitive unit cell of the Gd_2_MoO_6_ crystal in a monoclinic symmetry structure with the *C*_2_/*c*-space group view along the axis *c* and axis *b*.

### 4.2 Morphological Analyses

SEM-EDS measures were employed to characterize the morphology and elemental composition of the Gd_2_MoO_6_ compound. The SEM image revealed a nonuniform distribution of irregular format and some larger surface grains, as seen in Figure 3a,b. Also, it is possible to observe grain sizes between 5 and 8 μm. Additionally, the EDS spectrum shown in Figure 3c confirmed the presence of all expected chemical elements. The characteristic peaks of Gd, Mo, and O were also clearly identified, as shown in Figure 3c. The elemental compositions determined by EDS for Gd_2_MoO_6_, presented in Table 1, suggest proximity to the theoretical stoichiometry expected for the compound.

**Figure 3:**
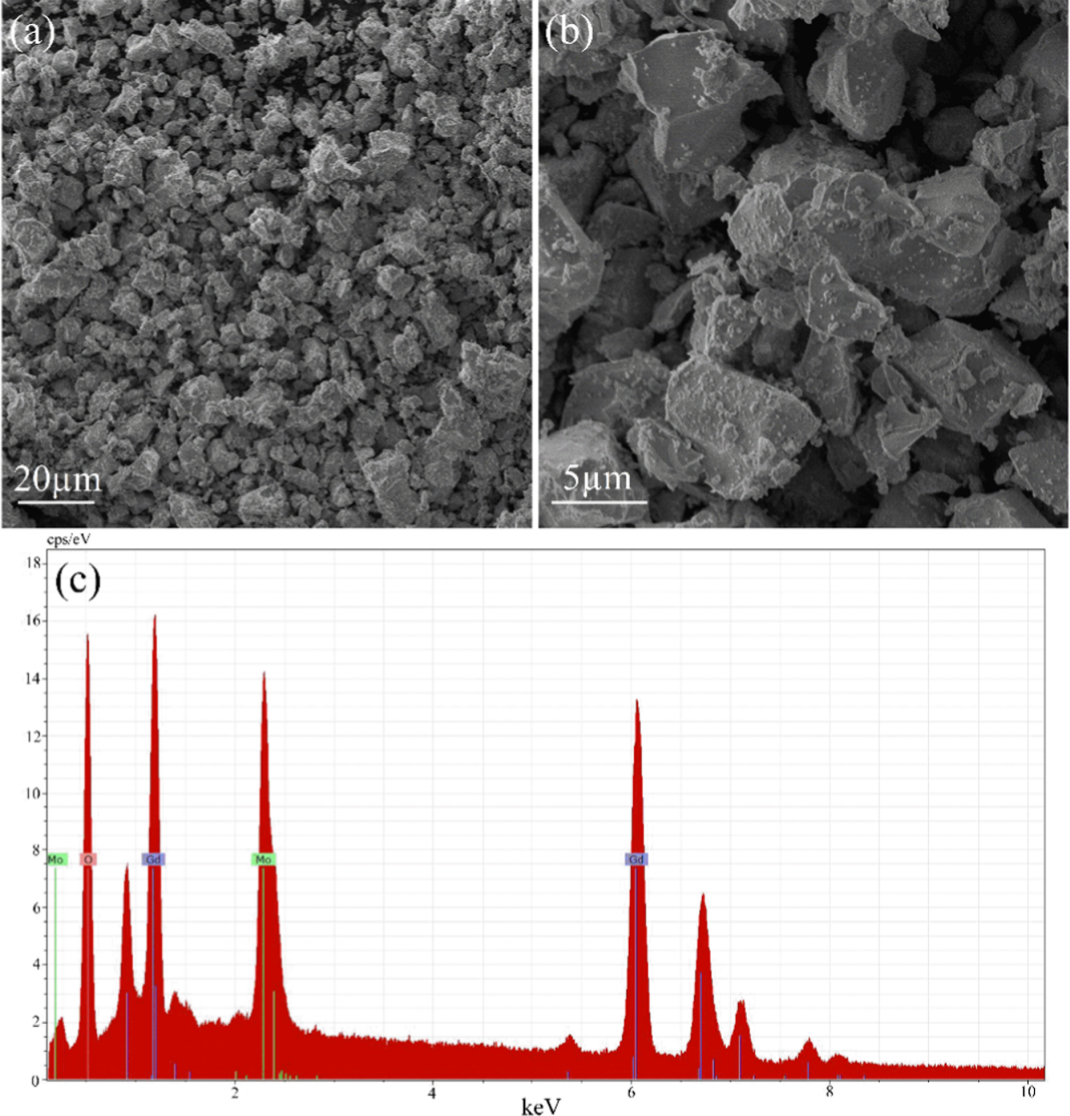
SEM micrographs of Gd_2_MoO_6_ polycrystalline samples: (a) image acquired at a scale of 20 μm, (b) higher magnification image with a scale of 5 μm. (c) EDS spectrum showing the presence of Gd, Mo, and O elements.

**Table 1:** Elemental Compositions of Gd_2_MoO_6_

element	weight %	atomic %	error (wt. %)
O	22.75	76.70	8.51
Mo	16.58	7.64	1.56
Gd	55.67	15.65	3.78
total	100	100	

### 4.3 Structural Features and Hirshfeld Surface Analysis

The primitive unit cell of Gd_2_MoO_6_, presented in Figure 4a, exhibits a well-ordered arrangement of Gd^3+^ (gray spheres), Mo^6+^ (green spheres), and O^2–^ (red spheres) ions, forming a complex framework of polyhedral coordination environments. The Gd^3+^ cations are typically surrounded by eight neighboring atoms in a square antiprismatic geometry, while Mo^6+^ occupies the center of MoO_5_ polyhedra, a characteristic motif in molybdates.^61^ Understanding the structure of this system in detail is critical for understanding the material stability and functional properties, as the strong ionic character of Gd–O bonds and the covalent nature of Mo–O bonds contribute to its structural and thermal resilience.

**Figure 4:**
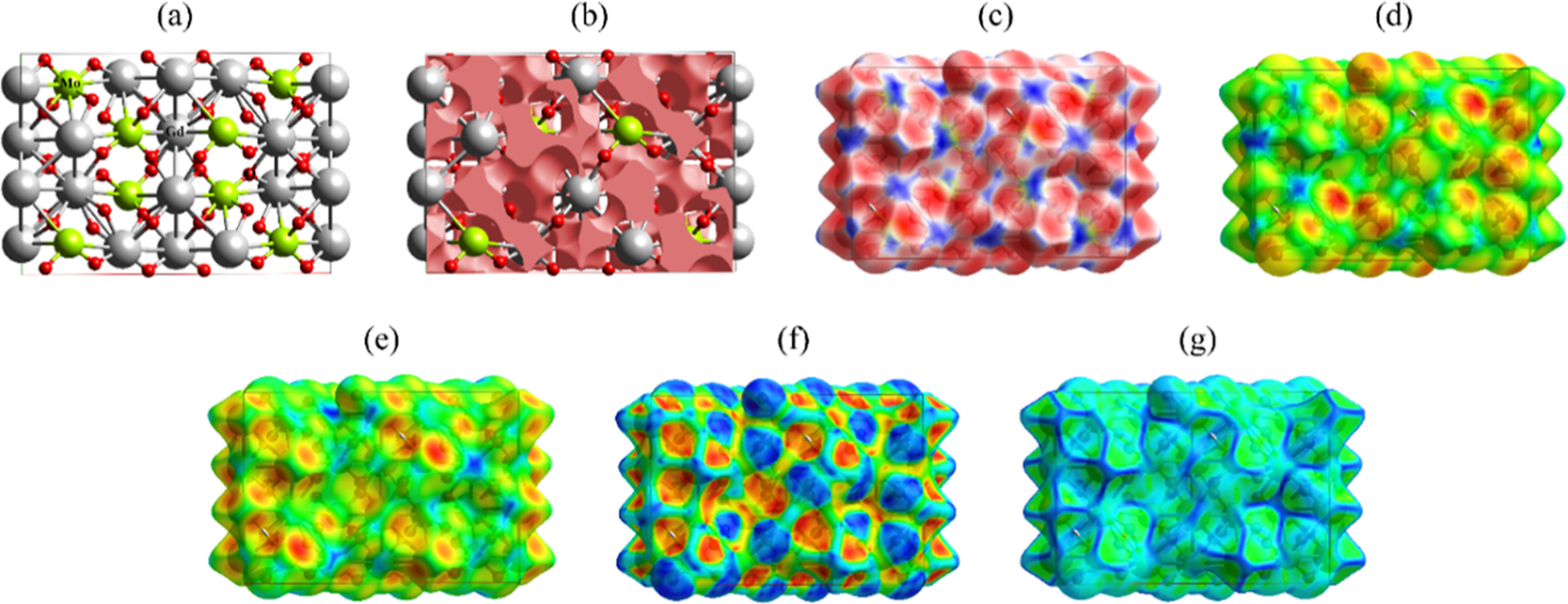
(a) Gd_2_MoO_6_ primitive unit cell. (b) Voids within the Gd_2_MoO_6_ structure visualized through isosurfaces (brown) along the *a*–*c* plane. Hirshfeld surfaces plots mapped according to (c) *d*_norm_, (d) *d*_e_, (e) *d*_*i*_, (f) shape index, and (g) curvedness.

Figure 4b shows the presence of voids (brown isosurfaces, 754.40 Å^2^) distributed along the *a*-*c* planes of the Gd_2_MoO_6_ primitive unit cell. These voids characterize regions of low electron density, originate from the packing inefficiencies inherent in the structure, or deliberate lattice under-coordination. Such voids act as diffusion channels for inserting external species into the structure with a free volume of 289.66 Å^3^ (30.4%). This percentage is considered high and suggests that great molecules or species with a large atomic radius can be incorporated into the lattice to optimize physicochemical properties of interest.^62,63^

The *d*_norm_ plot (Figure 4c), which normalizes contact distances based on *r*_vdW_, shows regions of strong contacts (red, predominantly around the O atoms) and weak contacts (blue). The asymmetric distribution of red spots near O atoms confirms the dominance of Gd–O/Gd–O and Mo–O/O–Mo bonds, which are primarily ionic and covalent, respectively. The presence of faint blue regions indicates areas where the packing is less dense, which correlates with the voids observed in Figure 4b.

The *d*_e_ map showed in Figure 4d reveals key intermolecular contacts through color-represented distances: red regions (≈1.2–1.5 Å) highlight close Gd–O interactions, yellow-green areas (≈1.6–2.2 Å) represent secondary Mo–O bonds, and blue zones (>2.5 Å) indicate weak Gd–Mo contacts (dispersive forces) or voids. In contrast, the *d*_*i*_ map (Figure 4e) probes internal bonding, with deep red (<1.0 Å) marking tight Gd–O coordination and orange-yellow (∼1.0–1.3 Å) reflecting covalent Mo–O bonds. The stark color contrast between Gd^3+^ (ionic, polarized electron density) and Mo^6+^ (covalent, localized bonds) underscores their distinct chemical roles. Furthermore, discrepancies in *d*_e_/*d*_*i*_ distributions near voids further identify low-density regions, offering targets for defect engineering in crystalline materials. These maps collectively validate the ionic-covalent hybrid nature of Gd_2_MoO_6_, with implications for tuning properties through controlled void manipulation.

The shape index map presented in Figure 4f reveals critical details about the surface topology through its color gradients. Red and orange regions correspond to concave areas (donor features), typically associated with O atoms accepting metal coordination sites. In contrast, blue zones represent convex regions (acceptor features), often marking protruding metal cations such as Gd^3+^ or Mo^6+^. The predominance of greenish hues indicates flat, neutral surfaces characteristic of extended Mo–O–Mo bridging lattices. Complementing this, the curvedness map (Figure 4g) displays distinct color regions that reveal important structural features. The dominant green areas represent the MoO_5_, indicating their rigid, covalent nature through moderate curvedness values (0.5–1.5 Å^–1^). These regions appear as smooth surfaces, reflecting the uniform electron density distribution characteristic of strong Mo–O covalent bonds. Surrounding these green zones, blue contours mark areas of gradual electron density transition, including the interfaces between MoO_5_ units and Gd coordination polyhedra as well as the boundaries of structural voids. This pattern confirms the hybrid ionic-covalent character of Gd_2_MoO_6_, where the MoO_5_ units maintain their structural stability, while the Gd–O regions allow for greater flexibility. The blue-outlined voids, showing the lowest curvedness values (<0.3 Å^–1^), identify potential sites for defect formation or ion migration pathways, which could be strategically exploited for crystal engineering.^64,65^

Figure 5 presents the 2D fingerprint plots of Gd_2_MoO_6_, which provide quantitative analyses of all intermolecular interactions. The symmetric distribution of the full fingerprint shows two prominent red spikes centered at d_i_, *d*_e_ low values, corresponding to the dominant short-range Gd···O/O···Gd and Mo···O/O···Mo bonds. These features confirm the dense, well-ordered nature of the crystal structure.

**Figure 5:**
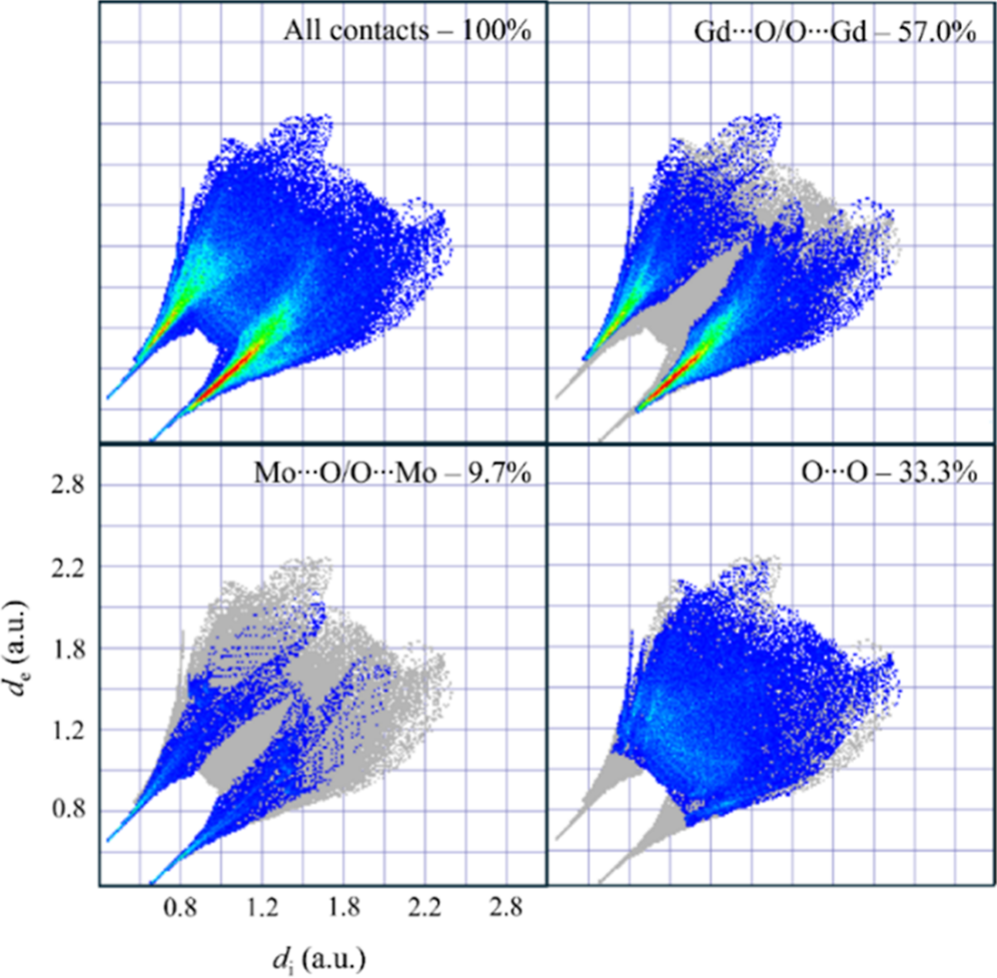
2D fingerprint graphs of the Gd_2_MoO_6_ system, displaying both overall and specific intermolecular contacts.

The decomposed 2D fingerprint plots reveal three distinct contact types: (i) Gd···O/O···Gd interactions (57.0%) dominate the fingerprint, indicating the ionic character of Gd–O bonding. The high density of these contacts indicates a tightly packed lattice with strong electrostatic cohesion, consistent with the material’s thermal stability and high melting point; (ii) Mo···O/O···Mo interactions (9.7%), which appear at shorter distances than Gd–O, highlighting the covalent nature of the MoO_5_ polyhedron. The narrow distribution of these contacts demonstrates the structural rigidity and bond-length uniformity of the Mo–O polyhedra; (iii) O···O interactions (33.3%) appear at intermediate distances, indicating a bridging oxygen lattice that supports structural integrity while enabling potential ion transport. The absence of metal–metal contacts confirms that electrostatic repulsion maintains cation dispersion. This fingerprint profile validates the Gd_2_MoO_6_ hybrid ionic-covalent bonding lattice.

### 4.4 Room-Temperature Structural and Vibrational Properties

The unit cell (Gd^3+^ ions at 4e and 8f sites, Mo^6+^ ions at 8f sites, and O atoms at 8f sites) comprises eight formula and the factor group analysis predicts 105 optical modes (*k* = 0) and 3 acoustic modes for the Gd_2_MoO_6_ crystal cell. The distribution of the optical modes is according to the irreducible representations of the factor group *C*_2*h*_ = 26A_g_ + 28B_g_ + 25A_u_ + 26B_u_, where A_u_ + 2B_u_ are acoustic modes. According to the selection rules, the A_g_ and B_g_ modes are Raman active, the A_u_ and B_u_ modes are IR active. Figure 6a,b shows the Raman and infrared spectra of polycrystalline Gd_2_MoO_6_ at room temperature, respectively. The number of vibrational modes theoretically predicted for the monoclinic phase exceed those observed experimentally, which can be attributed to the close energy proximity between some modes, the overlap of weak bands by stronger ones, and the limited resolution of the bands due to the random orientation of the crystallites within the sample. Furthermore, Table 2 shows the observed and calculated Raman and infrared modes, together with their assignments based on DFT calculations for the monoclinic phase.

**Figure 6:**
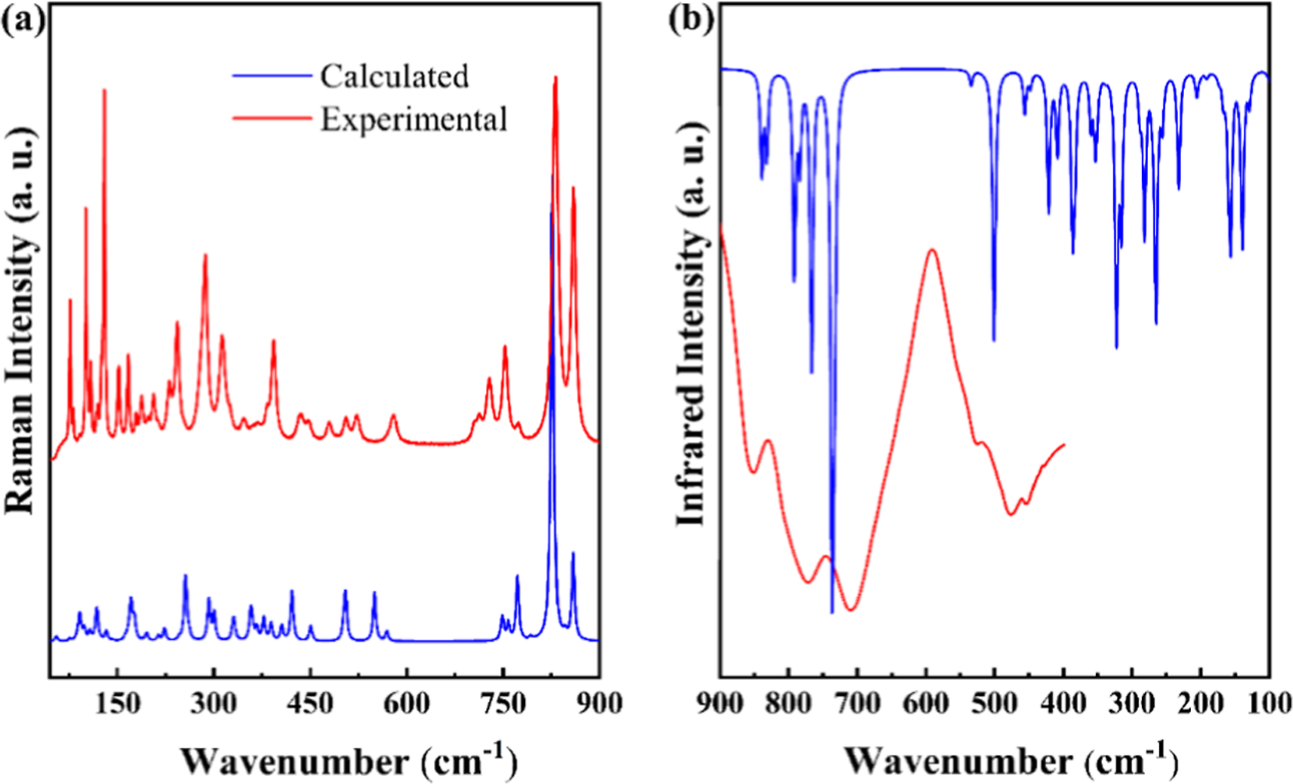
(a) Experimental and calculated Raman spectra of Gd_2_MoO_6_ in the 45–900 cm^–1^ region; (b) experimental and calculated IR spectra of Gd_2_MoO_6_ in the 900–100 cm^–1^ region.

**Table 2:** Analysis of Vibration Modes for the Gd_2_MoO_6_: Experimental Raman Modes (ω_R_), Experimental IR Modes (ω_IR_), Calculated Wavenumbers (ω_cal_), Irreducible Representations (Irrep.), and their Assignments^a^

ω_R_ (cm^–1^)	ω_IR_ (cm^–1^)	ω_cal_ (cm^–1^)	irrep	assignments^†^	ω_R_ (cm^–1^)	ω_IR_ (cm^–1^)	ω_cal_ (cm^–1^)	irrep	assignments
		52	B_g_	T_*y*_ [MoO_5_ + GdO_8_]	321		300	A_g_	δ [MoO_5_ + GdO_8_]
		55	B_u_	T [MoO_5_] + T_*xy*_ [GdO_8_]			316	B_u_	Sc [MoO_5_] + δ [GdO_8_]
76		76	B_g_	T [MoO_5_] + T_*x*_ [GdO_8_]			322	A_u_	δ [MoO_5_ + GdO_8_]
81		86	B_g_	T [MoO_5_ + GdO_8_]			330	B_g_	δ [MoO_5_ + GdO_8_]
91		91	A_g_	T [MoO_5_ + GdO_8_]	346		332	A_g_	δ [MoO_5_ + GdO_8_]
		93	A_u_	T [MoO_5_ + GdO_8_]			339	A_u_	δ [MoO_5_ + GdO_8_]
		96	B_u_	T [MoO_5_ + GdO_8_]	360		340	B_g_	δ [MoO_5_ + GdO_8_]
101		98	A_g_	T_*y*_ [MoO_5_] + T [GdO_8_]			350	B_u_	δ [MoO_5_] + tw [GdO_8_] in Gd–O–Gd bonds
		98	B_u_	T [MoO_5_ + GdO_8_]			353	A_u_	Lib [MoO_5_] + δ [GdO_8_]
		103	A_u_	T [MoO_5_ + GdO_8_]			357	B_g_	δ [MoO_5_ + GdO_8_]
107		108	A_g_	T [MoO_5_ + GdO_8_]			358	A_g_	δ [MoO_5_ + GdO_8_] + balance Gd–O
119		116	B_g_	T [MoO_5_ + GdO_8_]	367		359	B_g_	δ [MoO_5_ + GdO_8_] + balance Gd–O
125		118	A_g_	T [MoO_5_ + GdO_8_]			360	B_u_	Sc [MoO_5_] + δ [GdO_8_]
129		123	B_g_	T [MoO_5_ + GdO_8_]			367	B_g_	Sc [MoO_5_] + δ [GdO_8_]
		126	A_u_	T [MoO_5_ + GdO_8_]	383		378	A_g_	δ [MoO_5_ + GdO_8_]
		131	B_u_	T [MoO_5_ + GdO_8_]			386	A_u_	δ [MoO_5_ + GdO_8_]
		132	A_g_	T [MoO_5_ + GdO_8_]			387	B_u_	wag [GdO_8_] in Gd–O–Gd bonds
152		133	B_g_	T_*xy*_ [MoO_5_] + T_*x*_ [GdO_8_]	393		389	A_g_	δ [MoO_5_ + GdO_8_]
		140	B_u_	T [MoO_5_ + GdO_8_]	435		406	B_g_	δ [MoO_5_ + GdO_8_]
167		143	B_g_	T [MoO_5_ + GdO_8_]		425	407	B_u_	δ [MoO_5_ + GdO_8_]
		143	B_u_	T [MoO_5_ + GdO_8_]			418	A_u_	δ [MoO_5_ + GdO_8_]
		144	A_u_	T [MoO_5_ + GdO_8_]			418	B_g_	δ [MoO_5_ + GdO_8_]
		147	A_u_	T [MoO_5_ + GdO_8_]	446		421	A_g_	δ [MoO_5_ + GdO_8_]
		157	B_u_	wag [GdO_8_] in Gd–O–Gd bonds		449	422	B_u_	Lib [GdO_8_]
		160	A_u_	T [MoO_5_ + GdO_8_]			443	B_u_	δ [MoO_5_ + GdO_8_]
178		162	A_g_	Lib [MoO_5_] + δ [GdO_8_]			448	A_g_	δ [MoO_5_ + GdO_8_]
188		167	B_g_	T [MoO_5_ + GdO_8_]	479		451	B_g_	δ [MoO_5_ + GdO_8_]
		167	A_u_	T [MoO_5_] + Sc [GdO_8_]		476	455	A_u_	δ [MoO_5_ + GdO_8_]
197		171	A_g_	T_*y*_ [MoO_5_ + GdO_8_]			499	B_u_	δ [MoO_5_] + ρ [GdO_8_]
		174	B_u_	T [MoO_5_ + GdO_8_]			500	A_u_	Sc [MoO_5_] + δ [GdO_8_]
		176	B_g_	T [MoO_5_] + wag [GdO_8_]			501	B_g_	δ [GdO_8_]
206		177	A_g_	T [MoO_5_] + wag [GdO_8_]	505		505	A_g_	Motion of O atoms of the Gd–O bond along the *xy* diagonal
		190	B_u_	T [MoO_5_ + GdO_8_]		521	538	A_u_	strong motion of O atoms along the *x*-axisof the Gd–O–Gd bonds
		191	B_g_	δ [MoO_5_ + GdO_8_]	522		550	A_g_	Sc [GdO_8_], strong motion of O atoms in the Gd–O–Gd bonds
		192	A_u_	δ [MoO_5_] + wag [GdO_8_] in Gd–O–Gd Bonds	579		569	B_g_	ν [Gd–O]
212		195	A_g_	δ [MoO_5_] + T_*y*_ [GdO_8_]		556	594	A_u_	Sc [GdO_8_]
		200	B_u_	Lib [MoO_5_] + T [GdO_8_]			733	A_u_	ν_as_ [MoO_5_] + wag [GdO_8_] in Gd–O–Gd bonds
		205	A_u_	δ [MoO_5_ + GdO_8_]		705	736	B_u_	ν_as_ [MoO_5_] + wag [GdO_8_] in Gd–O–Gd bonds
		206	B_u_	δ [MoO_5_ + GdO_8_]	706		747	A_g_	ν_as_ [MoO_5_]
		212	B_g_	δ [MoO_5_ + GdO_8_]	713		749	B_g_	ν_as_ [MoO_5_]
231		214	A_g_	Lib [MoO_5_] + δ [GdO_8_]			753	A_u_	ν_as_ [MoO_5_]
		223	B_g_	δ [MoO_5_ + GdO_8_]	728		758	B_g_	ν_as_ [MoO_5_]
		232	A_u_	δ [MoO_5_ + GdO_8_]		771	766	B_u_	ν_as_ [MoO_5_]
243		246	A_g_	δ [MoO_5_ + GdO_8_]	752		772	A_g_	ν_as_ [MoO_5_]
		255	A_g_	δ [MoO_5_ + GdO_8_]			783	A_u_	ν_as_ [MoO_5_]
		256	B_u_	δ [MoO_5_ + GdO_8_]		786	791	B_u_	ν_as_ [MoO_5_]
284		261	B_g_	Lib [MoO_5_] + δ [GdO_8_]	773		793	B_g_	ν_as_ [MoO_5_]
		265	B_u_	δ [MoO_5_ + GdO_8_]			826	A_g_	ν_s_ [MoO_5_]
		270	A_u_	Lib [MoO_5_] + δ [GdO_8_]		801	831	B_u_	ν_s_ [MoO_5_]
		282	B_u_	δ [MoO_5_ + GdO_8_]		850	839	A_u_	ν_s_ [MoO_5_]
		288	A_u_	δ [MoO_5_ + GdO_8_]	832		846	B_g_	ν_s_ [MoO_5_]
		292	A_g_	Sc [MoO_5_ + GdO_8_]	859		859	A_g_	ν_s_ [MoO_5_]
312		293	B_g_	Lib [MoO_5_] + δ [GdO_8_]					

a T = translational; Sc = scissoring; Tw = twisting; Lib = librational; wag = wagging; ν = stretching; ρ = rocking; δ = bending; νas = anti- symmetric stretching; νs = symmetric stretching.

#### 4.4.1 Raman-Active Modes (A_g_ and B_g_ Representations)

To discuss the vibrational modes, we begin by examining the Raman-active modes. The Raman spectrum of the Gd_2_MoO_6_ crystal reveals vibrational modes associated with the A_g_ and B_g_ irreducible representations, which reflect structural deformations, polyhedral dynamics, and bonding characteristics. These modes are analyzed in three wavenumber regions for theoretical modes (in bold) with the corresponding experimental and some previously reported theoretical modes. A complete assignment is described in Table 2. (i) Low-wavenumber modes (50–200 cm^–1^): this region is primarily characterized by translational (T, T_*y*_, T_*x*_, T_*xy*_) modes, however, the appearance of the librational and bending modes of MoO_5_ and GdO_8_ is also evident. At 52 cm^–1^ (B_g_), the vibration corresponds to a translational (T_*y*_) motion of MoO_5_ and GdO_8_, indicating rigid-body translational interactions, illustrated in Figure S1. A similar translational movement is observed at **76 cm** ^**–1**^ **(B**_**g**_ **)**, with a translational (T) motion of MoO_5_ plus an additional T_*x*_ component from GdO_8_ contributes to the coupling effect. The mode at **86 cm** ^**–1**^ **(B**_**g**_ **)** and at **91 cm** ^**–1**^ **(A**_**g**_ **)** represents other translational vibration, this time with a broader influence on the T­[MoO_5_ + GdO_8_] units, while the mode at **98 cm** ^**–1**^ **(A**_**g**_ **)**, where the MoO_5_ and GdO_8_ polyhedra exhibit T_*y*_ and T motions, respectively, reflects the lattice dynamics. At **108­(A**_**g**_ **)**, **116 cm** ^**–1**^ **(B**_**g**_ **)**, **118 (A**_**g**_ **)**, and **123 cm** ^**–1**^ **(B**_**g**_ **)**, **143 cm** ^**–1**^ **(B**_**g**_ **)**, and **167 cm** ^**–1**^ **(B**_**g**_ **)**, the translational modes affect both polyhedral units. The **133 cm** ^**–1**^ **(B**_**g**_ **)** mode corresponds to T_*xy*_ translational vibrations of the [MoO_5_] polyhedra coupled with T_*x*_ motions of the [GdO_8_] polyedra. The mode at **162 cm** ^**–1**^ **(A**_**g**_ **)** involves a librational motion of the MoO_5_ units combined with bending (δ) vibrations of the GdO_8_ polyhedra, marking the onset of mixed external and internal vibrational character. The **A**_**g**_ modes at **171** cm^–1^ are associated with directional translational motion along the *y*-axis (T_*y*_) of the [MoO_5_ + GdO_8_] units, indicating anisotropic lattice dynamics governed by crystallographic orientation, while the **177 cm** ^**–1**^ **(A**_**g**_ **)** mode is attributed to translational vibrations of the [MoO_5_] units combined with wagging motions of the [GdO_8_] polyhedra. Finally, the **A**_**g**_ mode at **195 cm** ^**–1**^ is assigned to a bending (δ) vibration of the MoO_5_ polyhedra coupled with a directional translational motion along the *y*-axis (T_*y*_) of the GdO_8_ units. (ii) Midwavenumber modes (200–500 cm^–1^): encompassing bending (δ), librational (Lib), and scissoring (Sc) deformations, these modes predominantly influence Mo–O–Gd interactions. Bending and librational motions dominate this spectral region. The **214 cm** ^**–1**^ **(A**_**g**_ **)** mode arises from librational vibrations of the [MoO_5_] units together with bending (δ) motions of the [GdO_8_] polyhedra and for the mode at 223 cm^–1^ (B_g_), the vibration corresponds to a bending mode (δ) of MoO_5_ and GdO_8_, introducing distortions in the molybdate–gadolinium oxide interactions. This bending effect of polyhedral units is reinforced by the **246 cm** ^**–1**^ **(A**_**g**_ **)** and **255 cm** ^**–1**^ **(A**_**g**_ **)** modes, which indicate additional structural flexibility. A librational (Lib) mode of MoO_5_ appears at **261 cm** ^**–1**^ **(B**_**g**_ **)**, coupled with a bending (δ) motion of GdO_8_, while at 292 cm^–1^ (A_g_), a scissoring (Sc) mode from [MoO_5_ + GdO_8_] is also present and the mode at **293 cm** ^**–1**^ **(B**_**g**_ **)** is assigned to librational vibrations of the [MoO_5_] polyhedra coupled with bending (δ) motions of the [GdO_8_] units. The bending (δ) dynamic persists at **300 cm** ^**–1**^ **(A**_**g**_ **)**, **332 cm** ^**–1**^ **(A**_**g**_ **)**, **340 cm** ^**–1**^ **(B**_**g**_ **)**, **359 cm** ^**–1**^ **(B**_**g**_ **)**, **378 cm** ^**–1**^ **(A**_**g**_ **)**, **389 cm** ^**–1**^ **(A**_**g**_ **)**, **406 cm** ^**–1**^ **(B**_**g**_ **)**, **421 cm** ^**–1**^ **(A**_**g**_ **)**, and **451 cm** ^**–1**^ **(B**_**g**_ **)**, involving both polyhedral units. The mode at **359 cm** ^**–1**^ **(B**_**g**_ **)** exhibits a significant balance contribution from Gd–O bond bending, which is also observed in the other modes listed in Table 2 (iii) High-wavenumber Raman Modes (500–900 cm^–1^): in this region, the dominant modes are Mo–O stretching (ν_s_, symmetry; and ν_as_, antisymmetric) vibrations, which determine the rigidity and covalent character of Mo–O bonds. The mode centered at **505 cm** ^**–1**^ **(A**_**g**_ **)** is associated with the motion of oxygen atoms in the Gd–O bonds along the *xy* diagonal. The A_g_ mode at **550 cm** ^**–1**^ **(A**_**g**_ **)** is assigned to scissoring (Sc) of the GdO_8_ polyhedra and exhibits a strong displacement of oxygen atoms within the Gd–O–Gd bonds, confirms their structural role (illustrated in Figure S1). The **B**_**g**_ mode at **569 cm** ^**–1**^ corresponds to the stretching vibration (ν) of the Gd–O bond. The asymmetric stretching vibrations of the MoO_5_ units appear at **747 cm** ^**–1**^ **(A**_**g**_ **)**, **749 cm** ^**–1**^ **(B**_**g**_ **)**, **758 cm** ^**–1**^ **(B**_**g**_ **)**, **772 cm** ^**–1**^ **(A**_**g**_ **)**, and **793 cm** ^**–1**^ **(B**_**g**_ **)**, while the symmetric stretching modes are located at **846 cm** ^**–1**^ **(B**_**g**_ **)** and **859 cm** ^**–1**^ **(A**_**g**_ **)**.

#### 4.4.2 Infrared-Active Modes (A_u_ and B_u_ representations)

The IR spectra of the Gd_2_MoO_6_ system exhibits vibrational modes associated with the A_u_ and B_u_ symmetrical species, which correspond to dipole-active vibrations within the structure. These modes are classified according to their spectral domains and vibrational assignments. Regarding the (i) Low-wavenumber Region (50–200 cm^–1^): this region primarily consists of translational (T, T_*xy*_, T_*x*_, T_*y*_), bending and librational (Lib) motions involving the MoO_5_ and GdO_8_ units. At 55 cm^–1^ (B_u_), a translational motion (T) of MoO_5_ is coupled with a T_*xy*_ mode of GdO_8_, indicating a relative movement between these two polyhedral units. The 93 cm^–1^ (A_u_), 96 cm^–1^ (B_u_), 103 cm^–1^ (A_u_), 140 cm^–1^ (B_u_) and 143 cm^–1^ (B_u_) modes correspond to general translational (T) motions involving both MoO_5_ and GdO_8_, further confirming interactions between these polyhedra. The 157 cm^–1^ (B_u_) mode corresponds to a wagging motion (wag) of the GdO_8_ unit, specifically involving Gd–O–Gd bonds. The 167 cm^–1^ (A_u_) mode combines a translational motion (T) of MoO_5_ with a scissoring (Sc) mode of GdO_8_. (ii) Midwavenumber Region (200–500 cm^–1^): This region is dominated by librational (Lib), deformation (δ), and scissoring (Sc) vibrations, which involve internal distortions of MoO_5_ and GdO_8_. The modes at 205 cm^–1^ (A_u_), 206 cm^–1^ (B_u_), 232 cm^–1^ (A_u_), and 288 cm^–1^ (A_u_) are primarily assigned as the deformation (δ) vibrations of MoO_5_ and GdO_8_. The B_u_ mode at 316 cm^–1^ is assigned to a coupled vibration involving scissoring (Sc) of the MoO_5_ polyhedra and bending (δ) of the GdO_8_ units, while the modes observed at 322 cm^–1^ (A_u_) and 339 cm^–1^ (A_u_) are attributed to bending vibrations of both MoO_5_ and GdO_8_ polyhedra, indicating collective internal deformations. The B_u_ mode at 350 cm^–1^ corresponds to bending vibrations of the MoO_5_ units coupled with twisting (tw) motions of the GdO_8_ polyhedra, involving significant displacement within the Gd–O–Gd bonds. The B_u_ mode at 387 cm^–1^ is assigned to wagging vibrations of the GdO_8_ polyhedra, predominantly involving motions within the Gd–O–Gd bonding network. The modes at **407 cm** ^**–1**^ **(B**_**u**_ **)** and 418 cm^–1^ (A_u_) are attributed to bending vibrations involving both MoO_5_ and GdO_8_ polyhedra. Finally, the **B**_**u**_ mode at **422 cm** ^**–1**^ corresponds to a librational motion of the GdO_8_ units, while the modes at 443 cm^–1^ (B_u_) and **455 cm** ^**–1**^ **(A**_**u**_ **)** are assigned to bending vibrations of both MoO_5_ and GdO_8_ polyhedra, reflecting complex internal lattice dynamics. (iii) High-wavenumber Region (500–850 cm^–1^): this region is dominated by stretching (ν) vibrations, specifically symmetric (ν_s_) and antisymmetric (ν_as_) modes of MoO_5_ and GdO_8_. The **538 cm** ^**–1**^ **(A**_**u**_ **)** mode is associated with a strong vibration of oxygen atoms along the *x*-axis of the Gd–O–Gd bonds and the **A**_**u**_ mode at **594** cm^–1^ is attributed to scissoring of the GdO_8_ units. The **B**_**u**_ mode at **736 cm** ^**–1**^ involves asymmetric stretching of the MoO_5_ polyhedra (ν_as_) coupled with wagging motions of the GdO_8_ units within the Gd–O–Gd bonds. The asymmetric stretching vibrations of the MoO_5_ units are further observed at 753­(A_u_), **766 cm** ^**–1**^ **(B**_**u**_ **)** and **791 cm** ^**–1**^ **(B**_**u**_ **)**. The mode centers at **831 cm** ^**–1**^ **(B**_**u**_ **)** and **839 cm** ^**–1**^ **(A**_**u**_ **)** (illustrated in Figure S1) correspond to the symmetric stretching (ν_s_) of MoO_5_, representing the fundamental stretching dynamics of the polyhedral units.

### 4.5 Band Structure and PDOS

Figure 7 shows the band structure and PDOS. Figure 7a displays the calculated spin polarized band structure of Gd_2_MoO_6_ for spin up, Figure 7b spin down and Figure 7c calculated spin polarized PDOS of Gd_2_MoO_6_ by orbital. Analyzing the band structure is crucial for understanding the electronic properties of materials, as it provides essential insights into the energy levels and permissible electronic states within the crystal’s periodic lattice. This, in turn, sheds light on the conductivity, optical characteristics, and other key properties. Coulomb interaction with Hubbard *U* was incorporated with 4.38 eV for Mo 3*d* and 6 eV for Gd 4*f* orbitals according to Moore at al.^57^ The analysis reveals a band gap of 1.92 eV for the majority spin channel at the Gamma point, indicative of semiconducting behavior (Figure 7a). This gap is primarily attributed to the localized 4*f* orbitals of Gadolinium (Gd), a finding corroborated by the PDOS analysis presented in Figure 7c. PDOS analysis provides a detailed breakdown of the total density of states, resolving it into contributions from individual atoms and their respective orbitals. This enables a deeper understanding of the electronic structure and bonding characteristics within the material. In this context, PDOS indicates a significant contribution of the Gd 4*f* orbitals to the conduction band minimum states near the band gap, confirming their role in determining the observed semiconducting properties. Furthermore, the PDOS analysis reveals that the 3*d* orbitals of Molybdenum (Mo) atoms play a significant role in influencing the PDOS at conduction bands. This suggests that Mo 3*d* orbitals contribute to the overall electronic structure and may influence properties, such as conductivity or charge transport. The valence band states are dominated by the *p* orbitals of oxygen (O), having Mo *d*-orbitals and Gd *f*-orbitals a negligible contribution near the Fermi level. Figure 8 shows the PDOS for spin-up and spin-down electrons selected by the atoms. The Gd atom exhibits a noticeable distortion in this distribution, as shown in Figure 8a, suggesting the presence of magnetic character. In contrast, Figure 8b,c reveals relatively symmetrical distributions for the O and Mb atoms, respectively. This asymmetry in Gd PDOS can be indicative of magnetic interactions or localized magnetic moments. The overlap of Mo-3*d* with the states of the O-2*p* states suggests hybridization (Mo–O covalent bond). Gd *f*-orbital peaks likely appear as sharp, localized states deeper in the conduction band, having weak contributions near the Fermi energy level.

**Figure 7:**
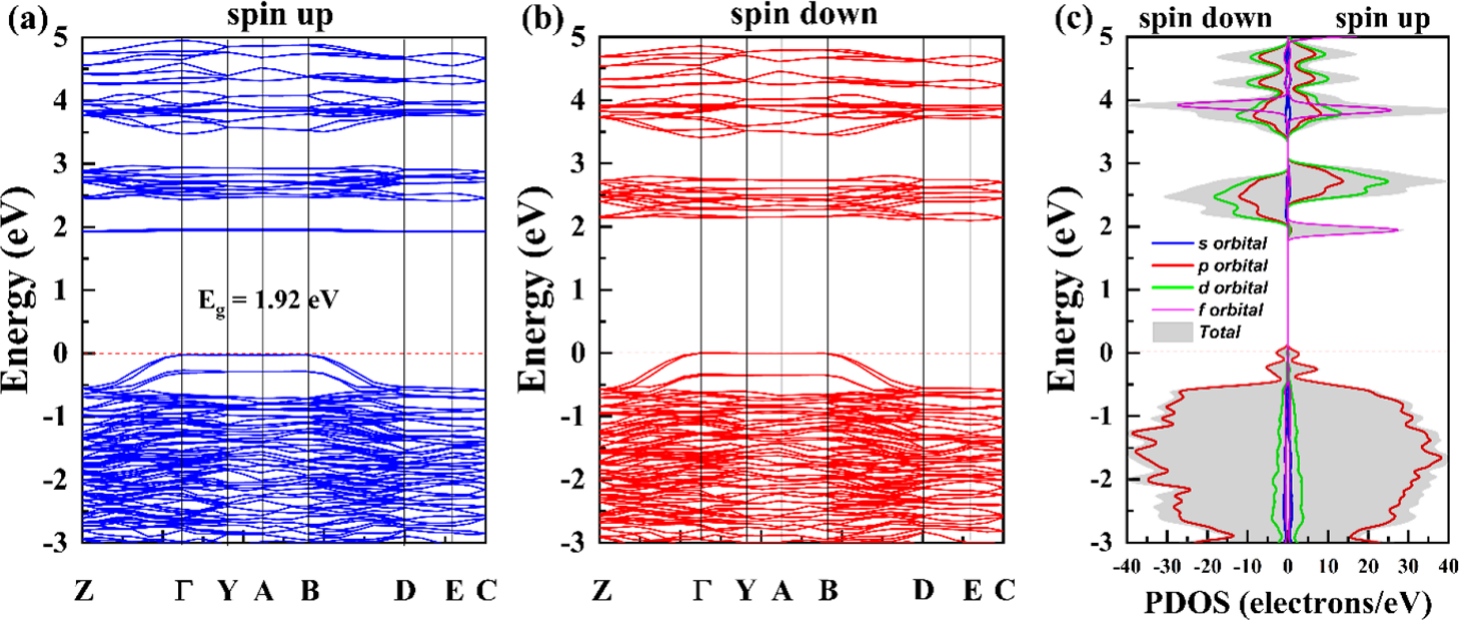
Calculated spin-polarized band structure of Gd_2_MoO_6_ for spin up (a) and spin down (b). (c) Calculated spin PDOS of Gd_2_MoO_6_ by orbital.

**Figure 8:**
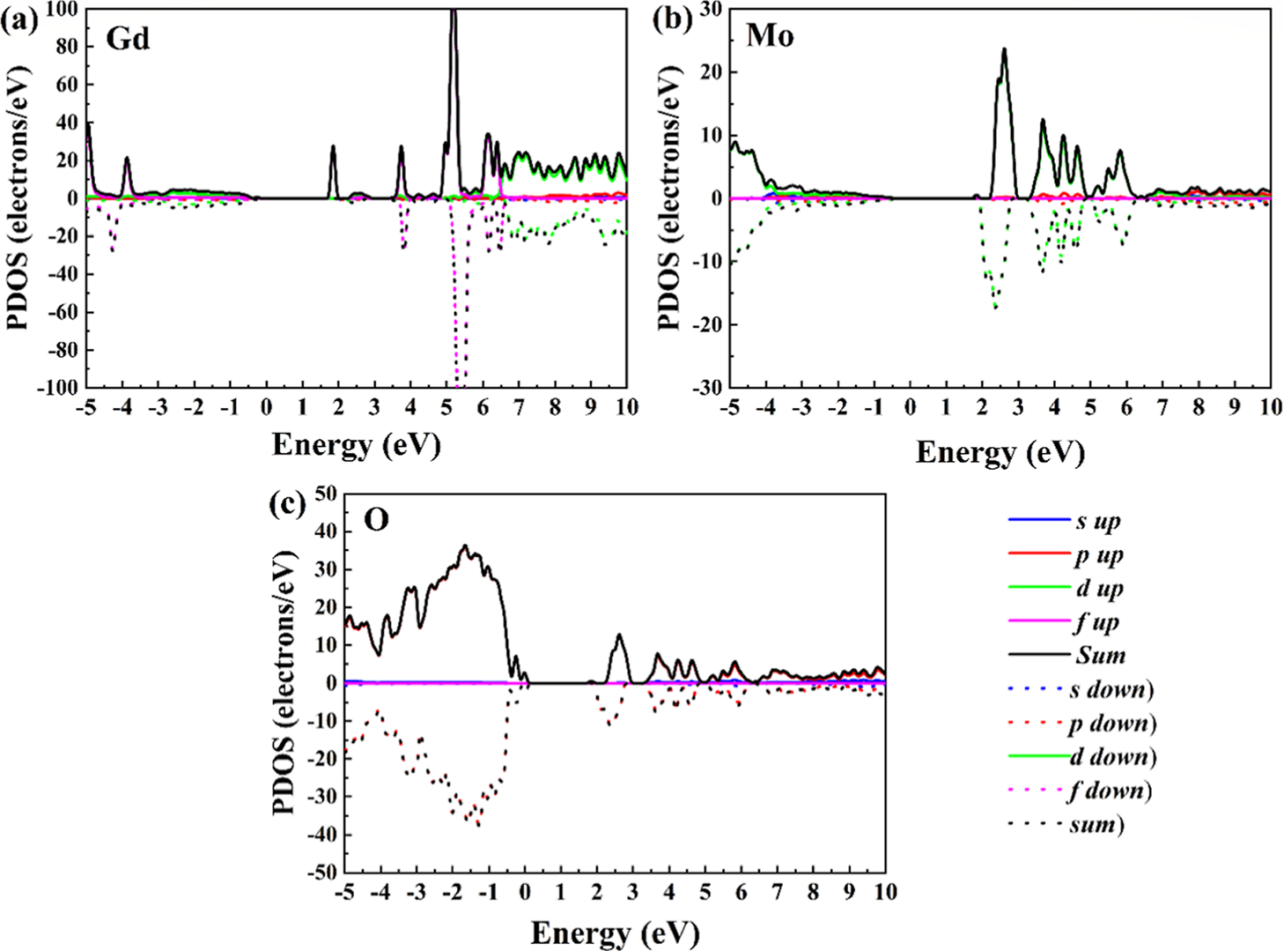
Calculated spin PDOS of Gd_2_MoO_6_ o selected by atom: (a) Gd, (b) Mo, and (c) O.

### 4.6 Pressure Dependence on the Vibrational Modes

Pressure serves as a critical parameter for probing the vibrational and structural properties of materials, particularly tungstates and molybdates, as extensively documented in the literature.^66–68^ High-pressure investigations aim to elucidate the alterations in octahedral units and the emergence of novel properties originating from such structural modifications. This way, an analysis of the Raman spectra of the Gd_2_MoO_6_ crystal under applied pressure, as presented in Figures 9 and 10, is furnished herein. As observed, there are some modifications in the Raman spectrum of the material, including the emergence, disappearance, and abrupt shifts in wavenumbers.

**Figure 9:**
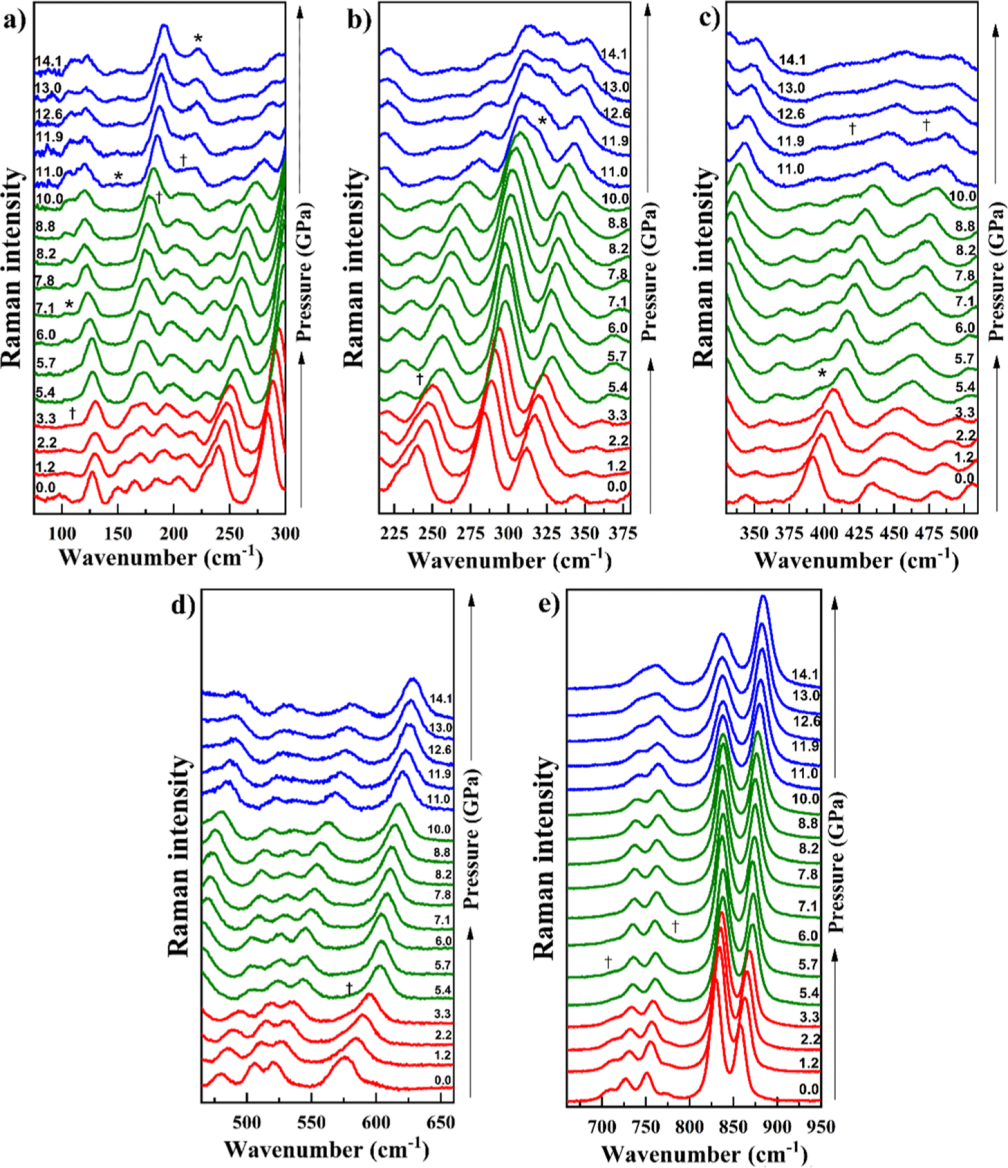
Pressure-dependent Raman spectra (0.01 to 14.1 GPa) of Gd_2_MoO_6_ crystals for the following spectral regions: (a) 75–300 cm^–1^, (b) 215–380 cm^–1^, (c) 330–510 cm^–1^, (d) 465–660 cm^–1^ and (e) 660–950 cm^–1^.

**Figure 10:**
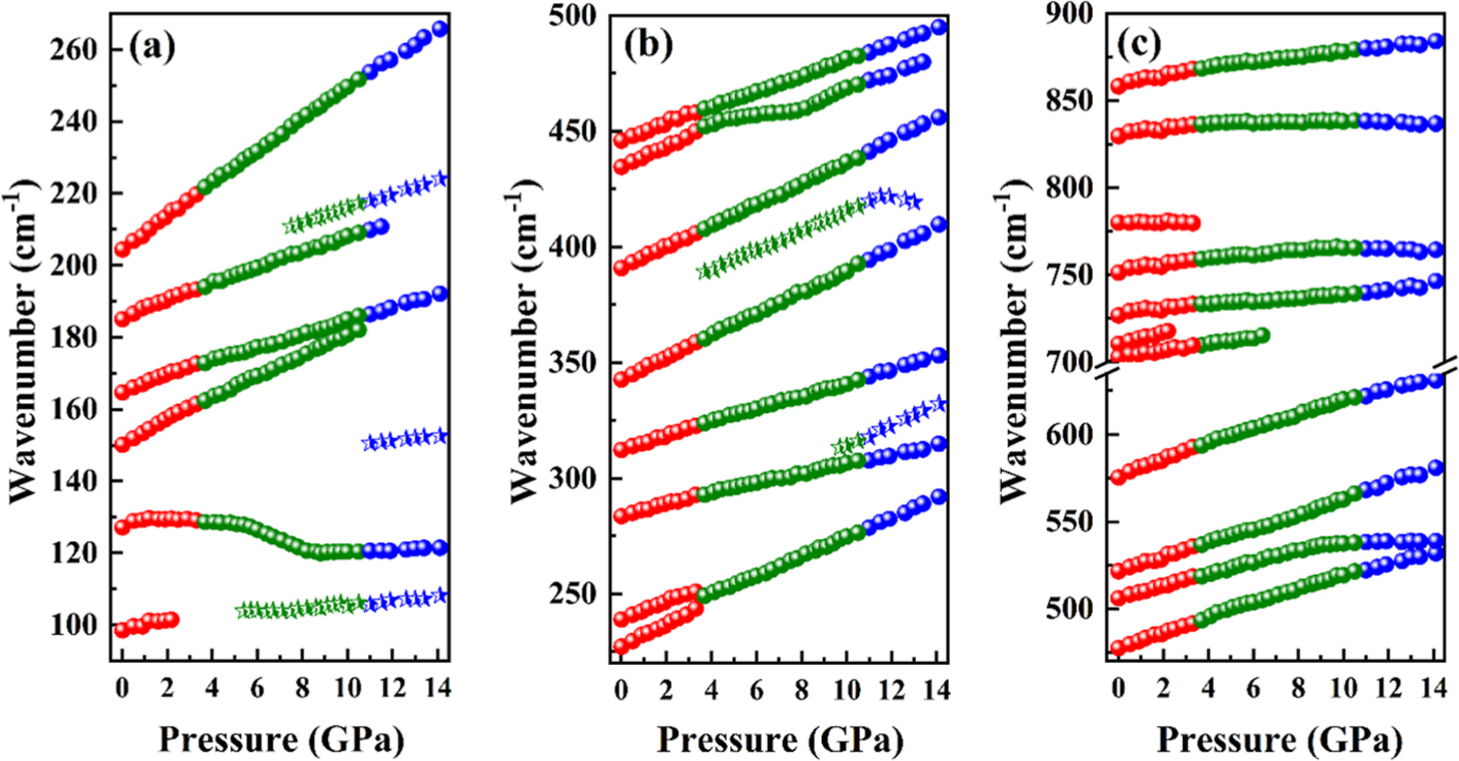
Wavenumber vs pressure plot (0.0 to 14.1 GPa) for the Raman modes of Gd_2_MoO_6_ crystals in the following spectral regions: (a) 90–270 cm^–1^, (b) 220–500 cm^–1^ and (c) 470–890 cm^–1^.

As previously discussed, this stated material is a complex molybdate with its Raman spectra governed by the vibrational modes of the MoO_5_ and GdO_8_ polyhedra and the Mo–O and Gd–O bonds. Overall, the intensities of the Raman modes decrease significantly at higher pressures (Figure 9). Regarding pressure dependence, the wavenumber of the Raman peaks generally increases with pressure (Figures 9 and 10). This is a common observation in high-pressure Raman spectroscopy, as the pressure compresses the bonds, leading to higher vibrational wavenumbers (blue shift). After starting running pressure, only one Raman mode, centered at 127 cm^–1^, exhibits wavenumber decreases with increasing temperature (dω/dP < 0), while all the other Raman modes exhibit wavenumber increases during compression (dω/dP > 0).

Figure 10 shows a plot of the relationship between wavenumber (cm^–1^) and pressure (GPa) for the Gd_2_MoO_6_. The figure is divided into three panels (a–c), each representing different regions of the Raman spectra under varying pressure conditions from 0.0 to 14.1 GPa in the following spectral regions: (a) 90–270 cm^–1^, (b) 220–500 cm^–1^, and (c) 470–890 cm^–1^. An interesting aspect observed for this material is that the pressure induces wavenumber shifts and discontinuities when the pressure increases up to 2.2 GPa, suggesting changes in the octahedral geometry and bonding environment (Figure 10). Above 2.2 GPa, the Gd_2_MoO_6_ system exhibits a few discontinuities and abrupt shifts in the wavenumber versus the pressure profile. Notably, the disappearance of a band near 100 cm^–1^ is observed, although its inherently low intensity renders the interpretation inconclusive. As such, it remains uncertain whether this mode, potentially a lattice vibration, is definitively suppressed at this specific pressure threshold. The additional spectral modifications observed at 2.2 GPa are relatively subtle; thus, they are unlikely to signify a structural phase transition. Instead, they may reflect a minor rearrangement within the crystal lattice.

However, concerning the modes emerging in the lattice mode spectral region, it is worth noting that the bands at 150 and 160 cm^–1^ converge in wavenumber, a process that persists up to approximately 10 GPa. Beyond this threshold, a single band is observed, suggesting the occurrence of a structural phase transition. Additionally, above 10 GPa, the splitting of a band near 300 cm^–1^ is observed with the newly emerged peak marked by an asterisk in Figure 9, along with a noticeable broadening of the bands in the 300–375 cm^–1^ range. Furthermore, the bands between 400 and 500 cm^–1^ become significantly broadened. As these features correspond to bending vibrations, the transition can be interpreted as indicative of an increase in disorder within the octahedral units.

It is noteworthy that above 5.4 GPa, certain changes are observed in the internal mode region such as the emergence of a band at 389 cm^–1^ (indicated by an asterisk) and the disappearance of a band near 710 cm^–1^ in the spectrum at 5.7 GPa. These alterations can be interpreted as structural accommodation under compression, although they cannot be attributed to a structural phase transition because there is no modification in the lattice mode region.

The peaks located at 98, 104, 151, and 165 cm^–1^ are likely associated with lattice vibrations involving external modes of the GdO_8_ and MoO_5_ polyhedrons. The emergence or disappearance of peaks within this region provides strong evidence for a first order phase transition in Gd_2_MoO_6_, notably marked at 10 GPa by the merging of two bands. A detailed peak-by-peak analysis reveals that the material undergoes significant changes in its vibrational properties under pressure, which can be ascribed to bond compression and structural transformation. Furthermore, previous investigations on the analogous compound Gd_2_(MoO_4_)_3_ indicate that the material undergoes several structural transitions between 2 and 6 GPa, followed by amorphization in the pressure ranges of 6 and 9 GPa. These transitions are clearly evidenced by pronounced changes of the Raman spectrum of the crystal, being associated with the continuous rearrangement and distortion of MoO_4_ tetrahedra and changes in molybdenum coordination, from IV to VI.^8,9,58^

### 4.7 The Principal Component Analysis (PCA) and Hierarchical Cluster Analysis (HCA)

The spectral variations observed during the compression process were analyzed through PCA Figure 11a and HCA Figure 11b, as illustrated in Figure 11. The HCA applied to the Raman spectra collected under pressures ranging from 0 to 14 GPa (Figure 11b) revealed a subdivision of the data into four distinct pressure intervals: 0–1.9 GPa, 2.2–6.7 GPa, 7.1–10.0 GPa, and 10.5–14.1 GPa. This grouping reflects modifications in the Raman signal intensity, which may be associated with the appearance or suppression of specific vibrational modes. A detailed analysis of these modes is necessary to better elucidate the physical processes underlying such transformations. It is noteworthy that the smallest distance between clusters was observed between the second and third groups, indicating that in this pressure range the spectra do not undergo abrupt modifications but rather subtle variations. In contrast, the other clusters exhibited distances greater than 1, which points to more pronounced and progressive changes in the spectral profiles. This behavior highlights that only in the region where the clusters are closer together is the spectral evolution smoother, whereas in the other ranges, the transformations occur more gradually across wider pressure intervals.

**Figure 11:**
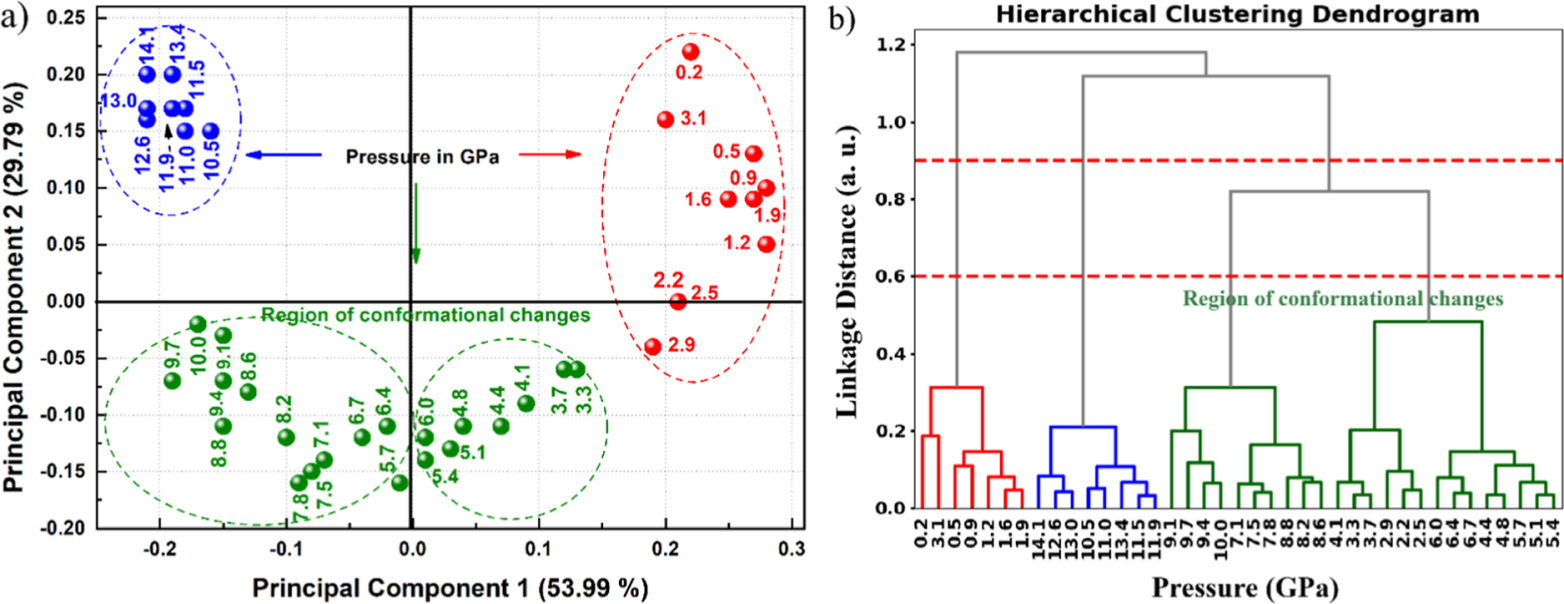
(a) PCA scatter plot (principal component 1 versus principal component 2) obtained from the high-pressure Raman spectra in the 75–950 cm^–1^ spectral range, together with the corresponding (b) HCA dendrogram, revealing four well-defined groups associated with distinct phase transformation regimes in Gd_2_MoO_6_.

Based on the PCA results, the spectra were grouped into three main clusters, with the transitions between them occurring around 3.1–3.3 and 10–10.5 GPa (Figure 11a). These transition intervals likely indicate changes in Raman spectral intensity, which may be associated with the disappearance of certain vibrational modes or with significant modifications in these pressure regions. These findings are consistent with the clustering patterns revealed by both PCA and HCA, which delineate regions of significant spectral change. The combined use of statistical and spectroscopic analyses enables a more comprehensive investigation of the physical phenomena responsible for these patterns. Such phenomena may include phase transitions, structural rearrangements, conformational modifications, or simply fluctuations in Raman signal intensity. Integrating these complementary approaches provides deeper insight into the mechanisms underlying the clustering behavior observed in the data set.^69–72^

In the present study on Gd_2_MoO_6_, however, two phase transitions were identified at near pressure ranges of 3.1–3.3 GPa and 9.5–10. GPa, which agree with the PCA analysis and HCA analysis. In particular, the stretching region of the MoO_5_ units displays the same number of vibrational modes throughout and evolves smoothly without any jump in wavenumber. Moreover, no evidence of amorphization was found, as all bands, particularly those corresponding to lattice modes, remain sharp and well-defined up to the highest pressure achieved in the experiment. Consequently, a clear distinction between the two compounds highlights the remarkable structural stability of Gd_2_MoO_6_ under compression. Naturally, further analysis using XRD data would be essential to elucidate the nature of the phase transition and characterize the high-pressure phase formed.

## 5 Conclusion

This study provides a comprehensive theoretical and experimental investigation of the electronic structure and vibrational properties of Gd_2_MoO_6_ under both ambient and high-pressure conditions. First-principles calculations reveal that Gd_2_MoO_6_ is a semiconductor with an indirect band gap of approximately 1.92 eV, with its conduction and valence band edges located at distinct high-symmetry points in the Brillouin zone. The vibrational properties, analyzed through DFT and high-pressure Raman spectroscopy, reveal significant pressure-induced changes. Gd_2_MoO_6_ shows a hybrid ionic-covalent lattice dominated by strong Gd–O and Mo–O bonds. The presence of voids and O···O contacts indicates structural flexibility and diffusion pathways, supporting its stability and potential for property tuning. The disappearance and emergence of specific Raman peaks, in particular associated with lattice mode, at a critical pressure range of 3.1–3.3 GPa and 9.5–10 GPa, indicates two structural phase transitions undergone by the material. PCA and HCA analyses identified two phase transitions in the pressure ranges of 3.1–3.3 GPa and 9.5–10.0 GPa, which are consistent with the pressure-dependent studies. These transitions suggest a reorganization of the Mo–O and Gd–O bonding environments, possibly involving changes in polyhedral connectivity. Nonetheless, future investigations incorporating XRD could provide deeper insights into the nature of this phase transition and confirm the high-pressure structural characteristics of the high-pressure phase of Gd_2_MoO_6_.

## Supplementary Material



## Notes

View of the calculated wavenumbers and related atomic displacements for some Raman and IR modes of Gd_2_MoO_6_ (PDF)
