# Supplementary material for: Electronic Structure, Lattice Dynamics, and Pressure-Induced Phase Transitions in Gd2MoO6: A Combined Theoretical and Experimental Study
Source: ACS Omega. 2026 Feb 10;11(7):12199–213. doi: 10.1021/acsomega.5c11574 (PMC12947017; doi:10.1021/acsomega.5c11574)
Supplement: Supplementary file 1 [file ao5c11574_si_001.pdf]

Supporting information

Electronic Structure, Lattice Dynamics, and Pressure-Induced Phase Transitions in Gd_2MoO_6: A Combined Theoretical and Experimental Study

Danilo S. Luz[a], Luiz F. L. da Silva[b], Raí F. Juca[c], Vicente O. Sousa Neto[c], Antônio J. Ramiro de Castro[d], Francisco F. de Sousa[e], Waldeci Paraguassu[e], Rômulo S. Silva[f], Lucas S.A. Olivier[f], José A. Lima Jr[f], Paulo de T. C. Freire[f], João G. de Oliveira Neto[a,*] and Gilberto D. Saraiva[a,c,*].

[a] Center for Social Sciences, Health, and Technology, Federal University ofMaranhão, Imperatriz, Maranhão 65900-410, Brazil

[b] Criminalistics Institute, Scientific Police of Pará, Marabá, Pará 68507-000, Brazil

[c] Faculty of Education, Sciences and Letters of the Sertão Central, State University of Ceará, Quixadá, Ceará 63902-098, Brazil;

[d] Federal University of Ceará, Cedro, Quixadá, Ceará 63902-580, Brazil

[e] Institute of Exact and Natural Sciences, Federal University of Para, Belém, Pará 66075-110, Brazil

[f] Department of Physics, Federal University of Ceará, Fortaleza, Ceará 60455-970, Brazil;

Corresponding authors: joao.gon@ufma.br (J. G. de Oliveira Neto)*; gilberto.saraiva@uece.br (G. D. Saraiva)**.

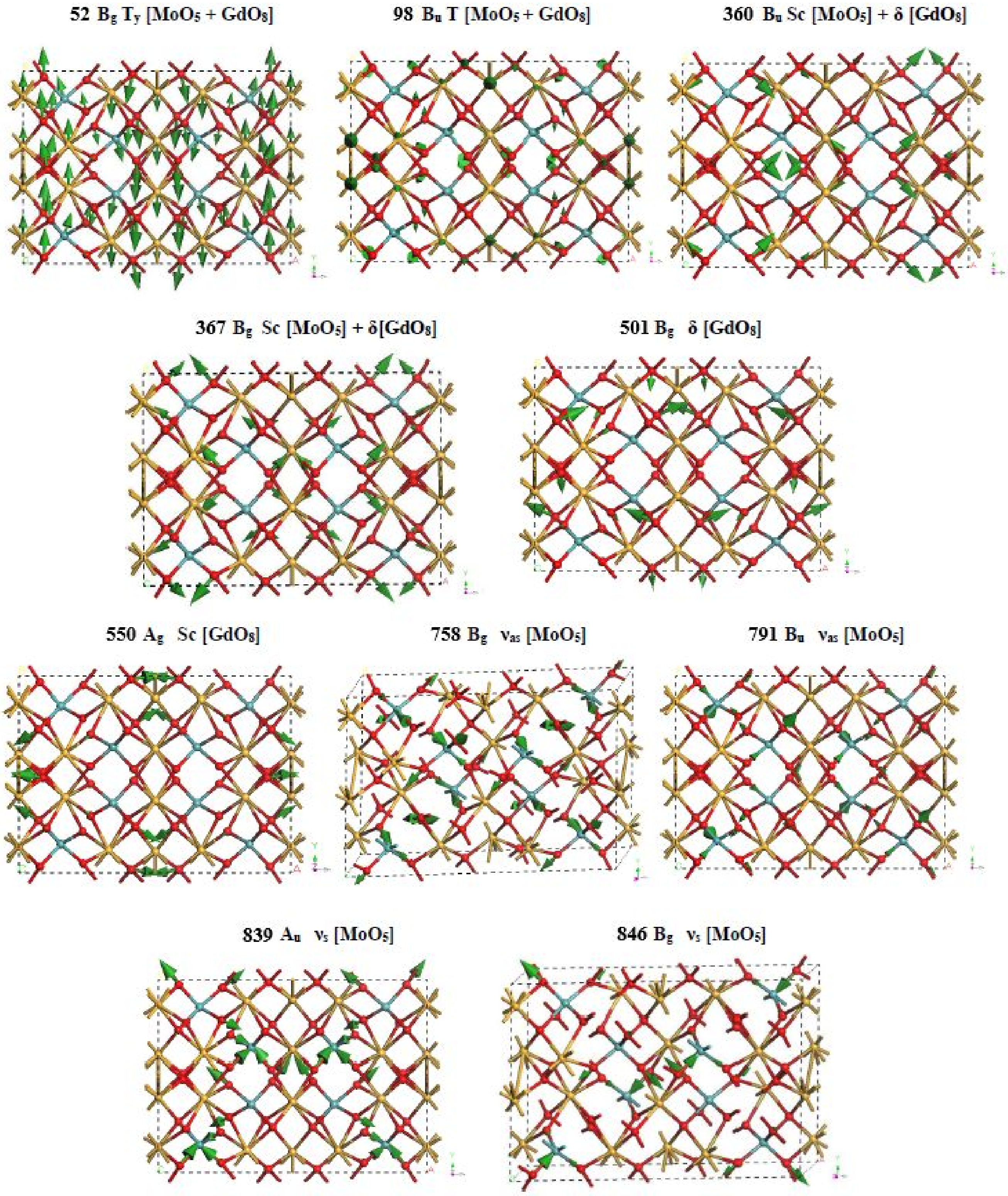

Fig. S1. Calculated wavenumbers and related atomic displacements for some Raman and IR modes of Gd_2MoO_6. The atomic displacements are seen along of the axe in the unit cell.
